# Pattern of triple negative epithelial ovarian cancer in indigenous African women ****

**DOI:** 10.12688/f1000research.9632.1

**Published:** 2016-09-28

**Authors:** Mustapha Akanji Ajani, Ayodeji Akeem Salami, Olutosin Alaba Awolude, Abideen Olayiwola Oluwasola

**Affiliations:** 1 Department of Pathology, University College Hospital, Ibadan, Nigeria; 2 Department of Obstetrics and Gynaecology, University College Hospital, Ibadan, Nigeria

**Keywords:** Estrogen Receptor, HER-2/neu expression, Immunohistochemistry, Ovarian carcinoma, Progesterone Receptor, Triple negative

## Abstract

**Background:** Triple negative epithelial ovarian cancer (TNEOC)  refers to ovarian carcinomas that do not express estrogen receptor (ER), progesterone receptor (PR) and human epidermal growth factor receptor- type 2 (HER-2/neu).  The aim of this study is to determine the pattern of triple negative epithelial ovarian cancer in indigenous African women.

**Methods**: We performed a retrospective review of ER, PR and HER-2/neu expression in 90 Nigerian patients with histologically diagnosed epithelial ovarian cancer. Lack of expression of ER, PR and HER2/neu antigens was used to determine carcinomas that are among the TNEOC. We also compared the clinicopathological parameters (age, International Federation of Gynaecology and Obstetrics (FIGO) stage, grade and histological subtype) in patients with TNEOC and non- TNEOC .

**Results: **Thirty-eight (42.2%) of the 90 tumours diagnosed as EOC were negative for ER, PR and HER2/neu expression. There was no significant association between TNEOC with other parameters such as age, FIGO stage and histological grade. Sixteen (66.7%) of the 24 mucinous carcinomas were triple negative, while only 21 (33.3%) of the 63 serous carcinomas were triple-negative and one (50%) of the two endometrioid carcinomas was triple negative. There was a significant association between triple-negative tumours and histological subtypes of EOC (p = 0.034).

**Conclusions**: A subtype of epithelial ovarian cancer that is negative for ER, PR and HER-2/neu has been discovered in indigenous African women. TNEOC expression is high and is comparable to the triple negative breast cancer subtype seen in people of African ancestry. Future study of TNEOC in a large sample size should be considered.

## Introduction

Epithelial ovarian cancer (EOC) remains one of the leading causes of death in gynaecological malignancies in developed countries ^1– 4^. The initial symptoms of ovarian cancer are often ambiguous, therefore it goes undiagnosed until after the disease is far advanced and has spread throughout the abdomen or to distant organs ^5, 6^.

Steroid hormone receptors expression in epithelial ovarian cancers have been proposed to have therapeutic and prognostic relevance, as is the case in breast cancers ^7^. The determination of tumour characteristics such as age, International Federation of Gynaecology and Obstetrics (FIGO) stage, grade and histological subtypes has been associated with clinical behaviour and impact on treatment and prognosis but have been found to be limited ^8^. Among the biological parameters proposed as possible prognostic factors in ovarian cancer, estrogen receptor (ER), progesterone receptor (PR) and human epidermal growth factor receptor- type 2 (HER-2/neu) have been tested as potential biomarkers that guide individualized treatment of the cancer ^5, 6, 9^. Epithelial ovarian carcinoma results from repeated ovulations, where the cumulative effects of each minor trauma on the ovarian epithelium can lead to malignant transformation ^10^. PR has been observed to predict better prognosis because of its protection against ovarian carcinoma development ^11, 12^. On the other hand, overexpression of ER has been found to be associated with poor prognosis due to its contribution to initiation and/or promotion of ovarian carcinogenesis ^10, 13^. The HER-2/neu has been shown to be over-expressed in approximately 20–30% of EOC with associated poor prognosis ^14– 16^.

Triple negative epithelial ovarian cancer (TNEOC) cases have been found to be more aggressive and display a worse prognosis than non-TNEOC cases ^17^. This was similarly observed in the studies of triple negative breast cancer ^18, 19^.

This study was designed to determine the pattern of TNEOC among indigenous African women and correlate it with clinicopathological parameters.

## Methods

### Patient selection

We performed a retrospective review of ER, PR and HER-2/neu expression in 90 patients with histologically diagnosed epithelial ovarian cancer seen at the University College Hospital, Ibadan, Nigeria between January 2006 and December 2012. Non-epithelial primary ovarian cancers and metastatic cancers in the ovary were not included in this study. The demographic data and clinical history of these cases were obtained from the case notes, surgical daybooks, surgical pathology request forms, post-mortem records and Cancer Registry data. Formalin-fixed paraffin-embedded tissue blocks of histologically diagnosed solid EOC between January 2006 and December 2012 were retrieved and used for the study. The microscopic grading (three-grade system) of Shimizu and Silverberg was used, which assesses architectural pattern, nuclear pleomorphism and mitotic activity ^20^. All histological classification of the EOC was based on the 2013 World Health Organisation (WHO) classification of ovarian tumours ^21^. The FIGO staging of the cases used for this study was extracted from the case notes of the patients.

### Ethics

The ethical clearance for this study was obtained from the Joint University of Ibadan/University College Hospital Ethical Review Committee (approval number UI/EC/13/0050) according to the Declaration of Helsinki.

### Immunohistochemistry

**Figure 1.**
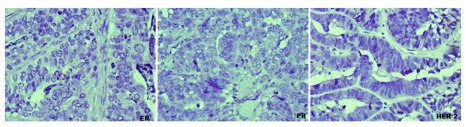
Photomicrographs showing ER (left), PR (middle) and HER-2/neu (right) negative expression (TNEOC) (immunostaining, 400X).

The immunostaining procedure for HER-2/neu was carried out in accordance with the previously published article ^22^. For the immunostaining procedure, three sections each for ER, PR and HER-2/neu at 5µm were cut from each of the paraffin-embedded tissue blocks after deparaffinization in xylene (two aliquots for five minutes each with the xylene covering the slide entirely). The sections were then rehydrated in graded alcohol concentrations (two aliquots each of 100% and 95% each and a single aliquot of 70%) in 250ml couplin jars. The antibodies used were monoclonal mouse anti-human ERα (Dako USA; clone ID5) and monoclonal mouse anti-human PR (Dako USA; clone PgR636) which identify the ER and PR nuclear protein antigens. The primary antibody used for HER-2/neu antigen was polyclonal rabbit anti-human C-erbB-2 (MBO/TEG, Dako USA, 1:800). The tissue sections were immersed in EDTA buffer (pH 9.0) for ER, citrate buffer (pH 6.0) for PR and in 1M Tris buffer (pH 9.0) for HER-2/neu. These slides were then incubated at room temperature for 20 minutes with primary monoclonal antibodies against ER (Dako USA, clone 1D5; 1:50), PR (Dako USA, clone PgR636; 1:50) and polyclonal rabbit anti-human C-erbB-2 (MBO/TEG, Dako USA, 1:800) followed by incubation in biotin-labelled secondary antibodies, polyclonal goat anti-mouse antibody for both ER and PR, (Dako USA, REF: K0675, LOT: 10081219) and polyclonal goat anti-rabbit antibody for HER-2/neu, (kitR, K5001, Dako Denmark) for 20 minutes and streptavidin-peroxidase complex (Dako USA, REF: K0675, LOT: 10084687) for another twenty minutes. The antigen-antibody complex was precipitated with di-aminobenzidine (DAB) for light microscopy with DAB substrate and DAB chromogen in the ratio of 1ml to 1 drop respectively. This was thereafter counterstained in Mayer’s haematoxylin (Dako USA). Dehydration of the sections was performed in ascending grades of alcohol and cleared in xylene. The slides were coversliped with DPX mountant. Known cases of breast cancer with positive reactions for ER, PR and HER-2/neu were used as a positive control. Negative controls were cases of tumour sections that were pretreated in Tris but without primary antibody immunostaining. All slides were reviewed independently by the three of the authors and cases with discordant scores were re-evaluated to have a consensus score. Grading of nuclear ER and PR staining was performed using an immunoreactive H-scoring system {none= 0 (negative); 1–25%=1+ (weak); 26–50%=2+ (moderate); >50%=3+(strong)} ^11^. HER-2/neu membrane staining was graded in according to the Hercep Test protocol system as 0, 1+, 2+ or 3+. Samples scored as 0 or 1+ were considered negative for HER-2/neu overexpression, 2+ was weakly positive and 3+ was strongly positive ^22, 23^. Photomicrographs of the specimens were taken using Olympus digital camera, DP 21 at 400X magnification ( Figure 1).

### Statistical analysis

The data obtained were subjected to statistical analysis using Statistical Package for Social Sciences (SPSS) version 20. Statistical analysis was used to evaluate statistical associations between TNEOC and clinicopathological parameters i.e. age, FIGO stage grade, and histological subtypes. Continuous variables were compared using the student’s T test and categorical variables were compared using the chi-square test, with the level of significance set at p <0.05.

## Results

Raw data for ‘Pattern of triple negative epithelial ovarian cancer in indigenous African women’ Click here for additional data file. Copyright: © 2016 Ajani MA et al. 2016 Data associated with the article are available under the terms of the Creative Commons Zero "No rights reserved" data waiver (CC0 1.0 Public domain dedication).

Thirty-eight (42.2%) of the 90 epithelial ovarian cancers (EOC) were negative for ER, PR and HER-2/neu expression ( Figure 1). There was no significant association between triple-negative EOC and age (p = 0.218), FIGO stage (p = 0.425) and histological grade (p= 0.269). There were more TNEOC cases seen in patients older than 40 years than those below 40 years of age. Of 38 cases of TNEOC, 21 (55.3%) were found in the early stage (FIGO stage I and II) of epithelial ovarian cancer and 17 (44.7%) were at the advanced stage.

However, sixteen (66.7%) of the 24 mucinous carcinomas were triple-negative, while only 21 (33.3%) of the 63 serous carcinomas were triple-negative and one (50%) of the two endometrioid carcinomas was triple-negative ( Table 1). There was therefore a significant association between triple-negative tumours and histological subtypes of EOC (p = 0.034).

**Table 1.** Correlation between triple negative epithelial ovarian cancer and clinicopathological parameters.

	TNEOC	Non-TNEOC	Total	P value
**Age**				
<40	9	4	13	
>40	29	48	77	0.218
**FIGO Stage**				
I-II	21	22	43	
III-IV	17	30	47	0.425
**Histological grade**				
I	10	9	19	
II	16	18	34	
III	12	25	37	
**Histological subtypes**				
Serous Carcinoma	21	42	63	
Mucinous Carcinoma	16	8	24	
Endometrioid Carcinoma	1	1	2	
Malignant Brenner tumour	0	1	1	0.034
**Total**	**38**	**52**	**90**	

## Discussion

A subgroup of epithelial ovarian cancer that is negative for ER, PR and HER-2/neu expression has been identified among indigenous African women. This subgroup is known as triple negative epithelial ovarian cancer (TNEOC). According to ER, PR, and HER-2/neu expressions, a breast cancer subtype known as triple negative breast cancer (TNBC) has been identified ^24^.

In our study, triple negative tumours accounted for 42.2% of EOC. This value contrasts with the results of other studies ^17, 25^ and compares with the results of previous study ^26^. A significant percentage (66.7%) of mucinous carcinoma were negative for ER, PR and HER-2/neu and this was statistically significant (p=0.034). This finding contrasts what was found from previous studies where there was no significant association between the TNEOC and histological subtypes ^17, 25, 26^. No significant association was also found between the TNEOC and histological grade unlike what was observed by Liu *et al*. ^17^ and de Toledo *et al*. ^26^ where TNEOC was significantly correlated with histological grade. There was no significant association between TNEOC and age and FIGO stage compared to the findings of other studies ^17, 25, 26^.

Our findings were comparable with what was found by Huo *et al*. in the population differences in breast cancer where triple negativity was predominant (27%) ^19^. In view of the fact that triple-negative breast cancers are more often seen in black Africans and African-Americans and are associated with a poorer prognosis than non-triple-negative breast cancers, further studies of TNEOC in different environments are required.

## Conclusions

A subtype of epithelial ovarian cancer that is negative for ER, PR and HER-2/neu has been discovered in Nigeria. Its (TNEOC) expression is high and is comparable to the triple negative breast cancer subtype seen in people of African ancestry. Future study of TNEOC in a large sample size should be considered.

## Data availability

The data referenced by this article are under copyright with the following copyright statement: Copyright: © 2016 Ajani MA et al.

Data associated with the article are available under the terms of the Creative Commons Zero "No rights reserved" data waiver (CC0 1.0 Public domain dedication). 

*F1000Research*: Dataset 1. Raw data for ‘Pattern of triple negative epithelial ovarian cancer in indigenous African women’, 10.5256/f1000research.9632.d136777 ^27^
