# Supplementary material for: Pattern of triple negative epithelial ovarian cancer in indigenous African women
Source: F1000Res. 2016 Sep 28;5:2415. [Version 1] doi: 10.12688/f1000research.9632.1 (PMC5089147; doi:10.12688/f1000research.9632.1)
Supplement: Peer review history

# Peer review: Referee response for version 1

**DOI:** 10.5256/f1000research.10379.r16697

**Published:** 2016-10-13

**Authors:** Saad Aliyu Ahmed

**Affiliations:** 1 Department of Pathology, Ahmadu Bello University Teaching Hospital (ABUTH), Zaria, Nigeria

1. This is an interesting manuscript, well conceived and has addressed the objective of the research.
2. The methodology is simple and reproducible.
3. Result was clearly outlined and discussion was adequate.
4. Conclusion is apt.

This research finding will add to the few literature on TNEOC in the population studied. It will serve as a baseline for future reference and interestingly the research has prognostic and therapeutic implications.

I have read this submission. I believe that I have an appropriate level of expertise to confirm that it is of an acceptable scientific standard.

# Peer review: Referee response for version 1

**DOI:** 10.5256/f1000research.10379.r16923

**Published:** 2016-10-11

**Authors:** Akinwumi Komolafe

**Affiliations:** 1 Department of Morbid Anatomy and Forensic Medicine, Obafemi Awolowo University, Ilé-Ifẹ̀, Nigeria

I have gone through the whole paper and these are my comments:

1. It is a well conceived and conducted research paper with the aims and objectives and rationale for the study well spelled out.
2. It addresses the peculiarities of the African race which is fundamental to adequate care.
3. The authors are very clear and consistent in the message that they aimed to convey and have passed this through.
4. All parts of the research work have fulfilled the guidelines for sound scientific work.
5. The discussion addresses crucial details.
6. The conclusion is a brilliant and excellent summation of the author's scientific work.

I have read this submission. I believe that I have an appropriate level of expertise to confirm that it is of an acceptable scientific standard.
